# Long non-coding RNA (lncRNA) plasmacytoma variant translocation 1 gene (PVT1) modulates the proliferation and apoptosis of acute lymphoblastic leukemia cells by sponging miR-486-5p

**DOI:** 10.1080/21655979.2022.2031405

**Published:** 2022-02-13

**Authors:** Jin-Ke Ju, Wei-Na Han, Cai-Ling Shi

**Affiliations:** a Department of Pediatrics, Changyi People’s Hospital, Changyi, China; b Department of General Surgery, Changyi People’s Hospital, Changyi, China

**Keywords:** PVT1, miR-486-5p, acute myeloid leukemia, MAML3

## Abstract

Long non-coding RNA (lncRNA) plasmacytoma variant translocation 1 gene (PVT1) is related to the progress of various cancers. Here, we illuminated the role of PVT1 in acute lymphoblastic leukemia (ALL) cell proliferation and apoptosis. PVT1 was upregulated in plasma samples from patients with ALL and ALL cell lines. PVT1 silencing repressed cell viability and enhanced cell apoptosis in Jurkat and SUP-B15 cells. PVT1 targeted microRNA-486-5p (miR-486-5p) and negatively modulated miR-486-5p expression. Upregulation of miR-486-5p decreased cell viability and increased ALL cell apoptosis. Mastermind Like Transcriptional Coactivator 3 (MAML3) was a downstream molecule of miR-486-5p and miR-486-5p mimic transfection weakened its expression in ALL cells. Rescue experiments proved that reintroduction of PVT1 counteracted the impacts of miR-486-5p in ALL cell proliferation and apoptosis. *In vivo*, PVT1 silencing repressed the tumor growth of SUP-B15 cells and reduced the expression of MAML3. Altogether, silencing of PVT1 inhibited ALL cell growth and induced cell apoptosis through sponging miR-486-5p.

## GRAPHICAL ABSTRACT

**Figure:**
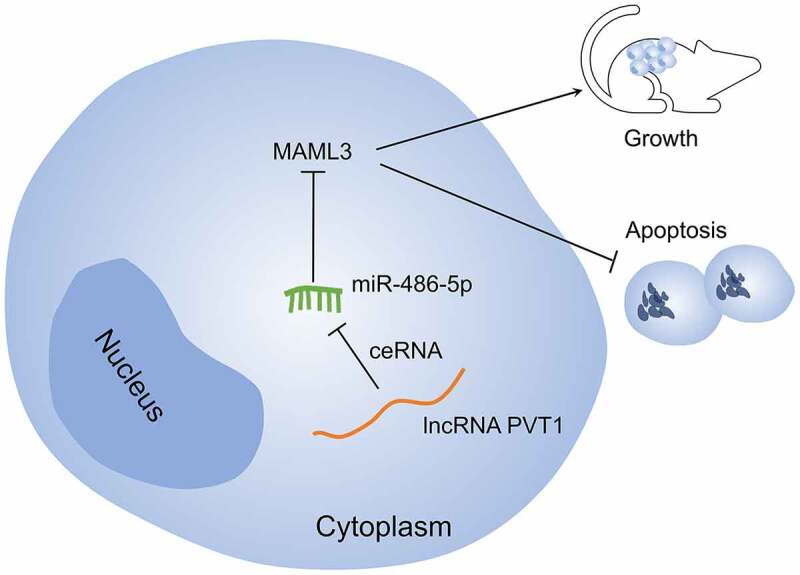

## Introduction

Despite the tremendous clinical progress in diagnostics and therapy for acute lymphoblastic leukemia (ALL), the prognosis of patients with refractory or relapsed ALL is unsatisfactory [1,2]. Hence, it is critical to investigate the signaling pathways underlying ALL pathogenesis and development to identify novel molecular targets for ALL treatment. LncRNAs exert core roles in the progression of malignant cancers, including ALL [3]. One of the first clinicopathological correlations with lncRNA expression data in ALL was performed by Fernando et al., who examined 160 children with B-ALL observing that lncRNA B-ALL associated long RNA-2 (BALR-2) correlates with overall survival and with response to prednisone [4]. Additionally, Garitano-Trojaola et al., while scrutinizing ALL samples and peripheral blood samples obtained from healthy donors, found 43 lncRNAs abnormally expressed in ALL. These genes may impact cancer traits such a cell proliferation, migration, apoptosis, and treatment response [5]. LncRNAs have substantial regulatory impacts in cancer development through acting as scaffolds to modulate the interactions between genes and proteins as decoys to bind miRNAs [6].

An early study manifests that the overexpression of PVT1 is related to the proliferation of leukemic cells in acute promyelocytic leukemia (APL) [7]. MiRNAs are a class of non-coding RNAs with about 18 ~ 25 nucleotides in length [8]. Increasing evidence demonstrated that miRNAs exert critical functions in several malignant cancers, including ALL [9]. MiR-486-5p has been identified to be downregulated in leukemia cells. Besides, miR-486-5p distinctly raises the cell apoptosis and caspase-3 activity in leukemia cells [10]. In addition, overexpressed miR-486-5p effectively inhibits non-small cell lung cancer (NSCLC) cell proliferation and invasion and successfully induces apoptosis *in vitro* [11]. The percentage of colorectal cancer cells undergoing apoptosis is raised after transfection with miR-486-5p mimic [12]. These results suggest that miR-486-5p is effective in inducing cells apoptosis in malignancies. Nevertheless, the precise mechanisms in lncRNA PVT1-miR-486-5p in ALL remain poorly understood.

In this study, we confirmed that lncRNA PVT1 was remarkedly higher in ALL. Then, we explored the functional activities of PVT1 on regulating ALL cell growth and apoptosis. Finally, we deliberated the practical implication of the PVT1-miR-486-5p axis in modulating ALL cell growth and apoptosis.

## Materials and methods

### Plasma specimens and cell culture

Plasma specimens were collected from 36 cases of ALL patients and 36 healthy controls from Changyi People’s Hospital (Shandong, China). Written consent was obtained from all participants and this study was approved by the Ethics Committee from the Changyi People’s Hospital (Approval No. 2017–0009A). ALL cells (Jurkat and SUP-B15) were bought from Nanjing Cobioer Biotechnology Co., Ltd (Nanjing, China), and human peripheral blood mononuclear cells (PBMC) cells were purchased from ATCC (Manassas, VA, USA). Jurkat and SUP-B15 cell lines were subjected to cell line authentication by short tandem repeat (STR) profiling. ALL cell lines were cultured in 1640 medium (Thermo Fisher Scientific) containing 10% fetal bovine serum (FBS) in 5% CO_2_ at 37°C. shRNA against PVT1 (sh-PVT1 #1 and #2) and negative control sh-NC, miR-486-5p mimics and negative controls (miR-NC) as well as pcDNA3.1-PVT1 and pcDNA3.1 vector were bought from GeneCopoeia (Guangzhou, China). shRNA or miRNA mimic was transfected into Jurkat or SUP-B15 cells using a Lipofectamine 2000 kit (Thermo Fisher Scientific) for 48 hours. The ALL cell lines were separately plated in 12-well plates and cultured in the incubator (Thermo Fisher Scientific, Waltham, MA, USA) for 24 h. The cells were transfected with MAML3 siRNA or nonspecific siRNA using Lipofectamine 2000 kit (Thermo Fisher Scientific) for 48 h.

### Quantitative real-time PCR (qRT-PCR)

Total RNAs were extracted using a miRNeasy extraction kit (QIAGEN). The miR-486-5p level was detected utilizing an All-in-one miRNA qPCR detection kit (GeneCopoeia, MD, USA). To detect PVT1 and MAML3, RNAs were extracted using TRIzol (Thermo Fisher Scientific) and transcribed into cDNA using All-in-One First-Strand cDNA Synthesis Kit (GeneCopoeia). qRT-PCR was carried out using an All-in-One qPCR mix (GeneCopoeia). U6 and glyceraldehyde-3-phosphate dehydrogenase (GAPDH) as the endogenous controls. The primers were shown as following: miR-486-5p, 5ʹ-ACACTCCAGCTGGGTCCTGTACTGAGCTGCCC-3ʹ (forward) and 5ʹ-CTCAACTGGTGTCGTGGAGTCGGCAATTCAGTTGAGCCCCGAG-3ʹ (reverse); U6, 5ʹ-CGACAAGACGATCCGGGTAAA-3ʹ (forward) and 5ʹ-GGTTGAGGAGTGGGTCGAAG-3ʹ (reverse); PVT1, 5ʹ-TGAGAACTGTCC TTACGTGACC-3ʹ (forward) and 5ʹ-AGAGCACCAAGACTGGCTCT-3ʹ (reverse); GAPDH, 5ʹ-GGAGCGAGATCCCTCCAAAAT-3ʹ (forward) and 5ʹ-GGCTGTTGTCATACTTCT CATGG-3ʹ (reverse); MAML3, 5ʹ-GAGGAT AGCTTCACCATCTTGC-3ʹ (forward) and 5ʹ-CCTCAGGAACCGTGTTGGC-3ʹ (reverse). The relative gene expression was calculated using the 2^−ΔΔCt^ method.

### CCK-8 assay

After transfection, Jurkat or SUP-B15 (2 × 10^3^ cells per well) were seeded into 96-well plates. Culture the cells in a CO_2_ incubator at 37°C for 24, 48, 72, or 96 h. Then, 10 μl of cell counting kit-8 (CCK-8) reagent (Bioworld, China) was placed into the plates. After incubation for three h, the absorbance in each well was quantified with a microplate reader BioTek Elx808 at 450 nm.

### Clonogenicity assay

1000 cells were incubated into six-well plates. Cells were fixed using 4% paraformaldehyde after 14 days and stained with 0.1% crystal violet. The number of visible colonies (>50 cells) in each group was calculated.

### Apoptosis assay

After 48 h of transfection, Jurkat or SUP-B15 cells in 600 μl of binding buffer were incubated with 5 μl of Annexin V-FITC reagent and Propidium Iodide (PI) reagent (Beyotime Biotechnology, China) for 15 mins. The proportion of apoptotic cells was determined using flow cytometry.

### Luciferase reporter assay

Segmental fragments of PVT1 or MAML3 3ʹ-UTR, including the binding sequences for miR-486-5p, were inserted into the pGL3 luciferase vector (Promega) to construct PVT1-wild type (wt) or MAML3-wt reporter, respectively. PVT1-mutant type (mut) or MAML3-mut reporter containing mutant binding sites was synthesized by GenePharma (Shanghai, China). The luciferase reporter combination with miR-486-5p was cotransfected into Jurkat or SUP-B15 cells using Lipofectamine 2000. Luciferase activity was assessed using a luciferase reporter assay system (Promega).

### Immunoblotting

Proteins were extracted using RIPA lysis (Beyotime Biotechnology). Equal amounts of proteins (30 μg) were separated using 8% SDS-PAGE and transferred into PVDF membranes (Millipore, Braunschweig, Germany). After incubation with MAML3 or β-tubulin primary antibody overnight at 4°C, the PVDF membrane was incubated with HRP-conjugated secondary antibody for 2 h. Finally, the bands were measured using an ECL kit (Millipore, Braunschweig, Germany).

### Xenograft experiment

100 μl of SUP-B15 cells that were transfected with sh-NC or sh-PVT1 (1 × 10^8^ cells/ml) were subcutaneously inoculated into BALB/c nude mice (n = 3 in each group). Xenograft tumor was assessed weekly. Tumor volume = (length × width^2^)/2 [13]. The animal experiment was conducted with the Guide for the Care and Use of Laboratory Animals (NIH publication). Five weeks post-injection, nude mice were sacrificed. The animal protocol has been approved by the Committee of the Ethics of Animal Experiments of the Changyi People’s Hospital.

### Statistical analysis

All data were shown as Mean ± SD from three individual experiments and calculated using GraphPad Prism 7. The difference was calculated using Student’s t-test or ANOVA post hoc Tukey’s test. The relevancy among PVT1, miR-486-5p, and MAML3 was measured using Pearson correlation analysis. *P*< 0.05 was statistically significant.

## Results

This work aimed to evaluate the expression pattern of lncRNA PVT1 in ALL and explore the potential effects of PVT1 in ALL cell growth and apoptosis. *In vitro* and *in vivo* assays, we found that deletion of PVT1 could suppress proliferation and promote apoptosis of ALL cells *via* sponging miR-486-5p and regulating MAML3.

### Silencing of PVT1 induces ALL cell apoptosis

**Figure 1.**
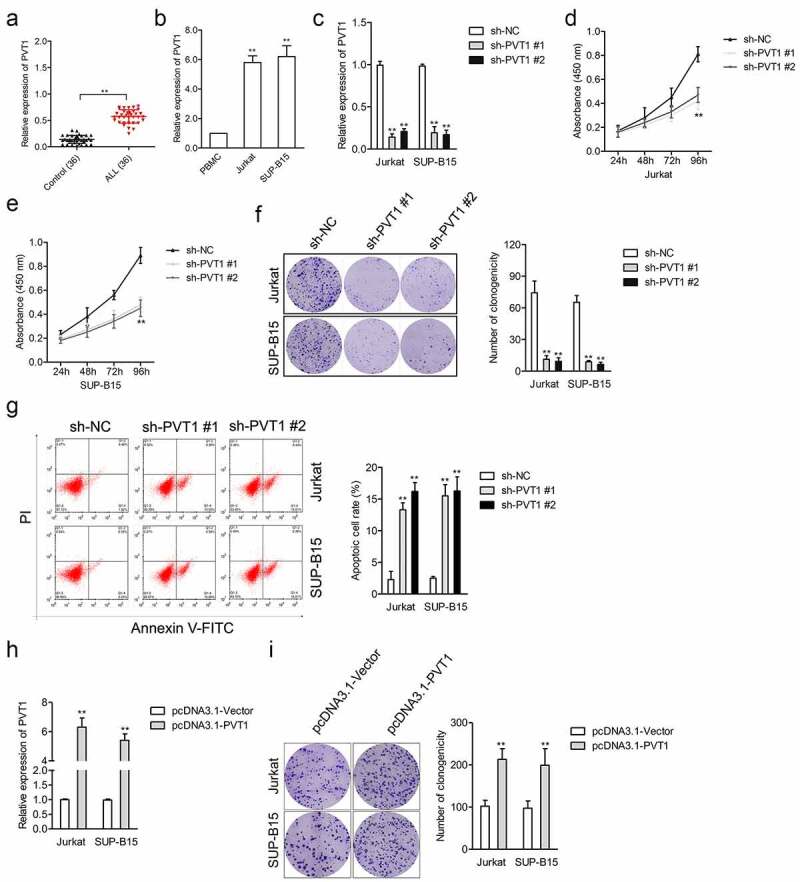
The upregulated expression level of PVT1 was observed in ALL. (a) qRT-PCR analysis for PVT1 expression level in plasma samples from ALL and healthy controls. **P< 0.01 compared to control. (b) PVT1 expression level in ALL Jurkat and SUP-B15, as well as PBMC. **P< 0.01 compared to PBMC. (c) Jurkat and SUP-B15 cells were transfected with sh-NC or sh-PVT1. The relative expression level of PVT1 after transfection was detected using the qRT-PCR assay. (d-e) The proliferation of Jurkat and SUP-B15 cells was detected by a CCK-8 assay. (f) Colony formation analysis of colony formation in Jurkat and SUP-B15 cells. (g) Cell apoptosis assay for transfected Jurkat and SUP-B15 cells. (h) Jurkat and SUP-B15 cells were transfected with pcDNA3.1-Vector or pcDNA3.1-PVT1. The relative expression level of PVT1 after transfection was detected using the qRT-PCR assay. (i) Colony formation analysis of colony formation in Jurkat and SUP-B15 cells. **P< 0.01 compared to pcDNA3.1-Vector.

Firstly, the levels of PVT1 were analyzed in plasma samples from patients with ALL and ALL cells. ALL cells originate from precursor lymphoid T- (T-ALL) and B-cells (B-ALL). Two cell lines, SUP-B15 and Jurkat cells, representing B-ALL and T-ALL, respectively, were used for the *in vitro* functional analysis [14,15]. The result of the qRT-PCR analysis showed that the PVT1 level was noticeably higher in plasma samples from ALL compared with that in the control group (Figure 1(a)). Meanwhile, ALL cells (Jurkat and SUP-B15) exhibited a significantly higher level of PVT1 than human PBMC (Figure 1(b)). shRNAs against PVT1 (sh-PVT1 #1 or sh-PVT1 #2) was transfected into Jurkat and SUP-B15 cell to construct PVT1 knockdown ALL cells to seek the impact of PVT1 on ALL cell proliferation and apoptosis. The result from qRT-PCR verified that sh-PVT1 transfection caused a significant decrease of PVT1 expression in Jurkat and SUP-B15 cells (Figure 1(c)). Then, the CCK-8 test indicated that PVT1 silencing induced a significant inhibition in the cell viability of Jurkat and SUP-B15 cells (Figures 1(d,e)). Moreover, the results of colony formation analysis suggested that the clonogenicity efficiency of sh-PVT1 transfected ALL cell was dramatically lower than that in the sh-NC group (Figure 1(f)). Cell apoptosis was increased in Jurkat and SUP-B15 cells upon treatment with sh-PVT1 (Figure 1(g)). Nevertheless, we proved that upregulation of PVT1 enhanced the colony formation of ALL cells *in vitro* (Figures 1(h,i)). All these findings indicated that silencing of PVT1 induced ALL cell apoptosis and inhibited cell growth.

### PVT1 serves as a ‘sponge’ for miR-744-3p

**Figure 2.**
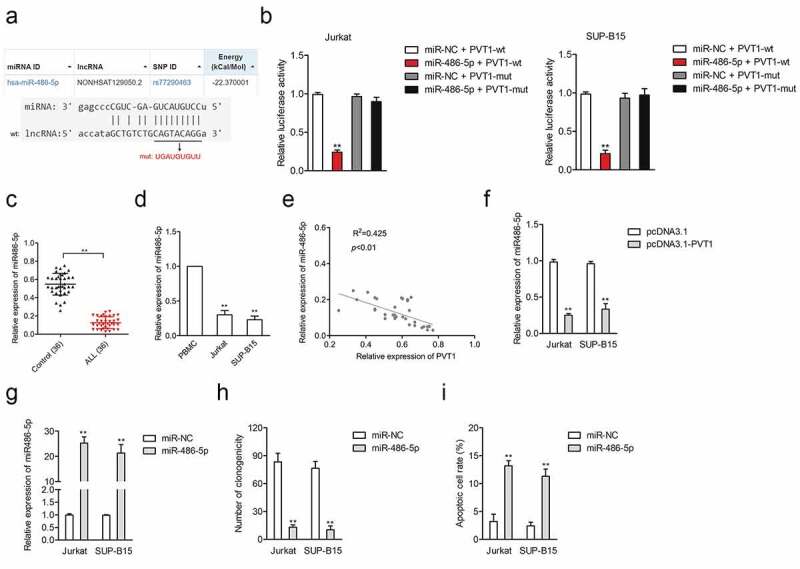
LncRNA PVT1 directly targeted miR-486-5p. (a) The predicted binding site of miR-486-5p and PVT1 and the mutant. (b) Luciferase reporter assays for Jurkat and SUP-B15 cells transfected with PVT1-wt reporter or PVT1-mut reporter, as well as miR-486-5p mimics. (c) The expression of miR-486-5p in plasma samples from ALL and healthy controls. (d) MiR-486-5p expression in ALL Jurkat and SUP-B15 cells, as well as PBMC. (e) Pearson correlation analysis for the correlation between relative expression levels of PVT1 and miR-486-5p in ALL. (f) qRT-PCR assay for miR-486-5p expression in Jurkat and SUP-B15 cells transfected with pcDNA3.1-PVT1 or pcDNA3.1. (g) Jurkat and SUP-B15 cells were transfected with miR-NC or miR-486-5p mimics. The relative expression level of miR-486-5p after transfection were detected using the qRT-PCR assay. (h) Colony formation analysis of colony formation in Jurkat and SUP-B15 cells. (i) Flow cytometry analysis of cell apoptosis in Jurkat and SUP-B15 cells. **P< 0.01 compared to sh-NC or pcDNA3.1.

Competing endogenous RNA (ceRNA) is essential in elucidating how lncRNAs act as a ‘sponge’ for miRNAs relative to cancer biology functions. Putative binding sites between miR-486-5p and PVT1 were predicted using the bioinformatics tool (LncBase Predicted v.2) and were shown in Figure 2(a). Next, PVT1-wild type (wt) luciferase reporter carrying the binding sites for miR-486-5p or PVT1-mutant type (mut) reporter containing the mutant pair sequences were transfected into Jurkat or SUP-B15 cell combination with miR-486-5p. In Figure 2(b), miR-486-5p reduced the luciferase activities in Jurkat and SUP-B15 cells transfected with PVT1-wt reporter, while it exhibited no impact on luciferase activities of PVT1-mut reporter. The levels of miR-486-5p in both ALL plasma samples and cells were evaluated using qRT-PCR. As presented in Figures 2(c,d), miR-486-5p was downregulated in the plasma specimens collected from patients with ALL and cells. The Pearson correlation assay showed that the PVT1 level was negatively associated with the miR-486-5p level (Figure 2(e)). Finally, we observed that upregulation of PVT1 after pcDNA3.1-PVT1 transfection declined miR-486-5p level in both SUP-B15 and Jurkat cells (Figure 2(f)). To explore the role of miR-486-5p in ALL, miR-486-5p mimic was transfected into Jurkat and SUP-B15 cells (Figure 2(g)). The outcomes of colony formation analysis exhibited that miR-486-5p restrained the growth of ALL cells in vitro (Supplementary Figure 1A and Figure 2(h)). Besides, apoptosis assay implied that miR-486-5p strikingly induced Jurkat and SUP-B15 cells apoptosis (Supplementary Figure S1B and Figure 2(i)). All these findings indicated the negative relationship between PVT1 level and miR-486-5p level in ALL.

### MAML3 is a target gene of miR-486-5p

**Figure 3.**
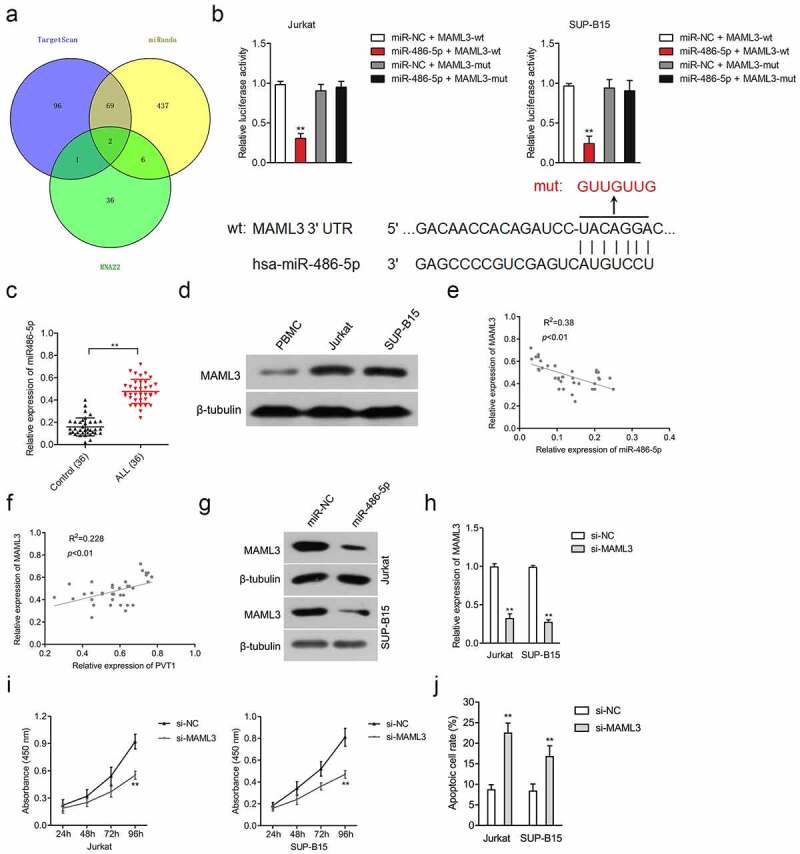
MiR-486-5p targeted and negatively regulated MAML3. (a) The putative binding site of miR-486-5p on 3ʹ-UTR of MAML3 was predicted by miRanda, RNA22, and TargetScan bioinformatics tools. (b) Luciferase reporter assays for Jurkat and SUP-B15 cells transfected with MAML3-wt reporter or MAML3-mut reporter, as well as miR-486-5p mimics. (c) The expression of MAML3 in plasma samples from ALL and healthy controls. (d) The expressions of MAML3 in Jurkat and SUP-B15 cells, as well as PBMC, were detected by Western blotting. (e-f) Pearson correlation analysis for the correlation between relative expression levels of MAML3 and PVT1, miR-486-5p in ALL. (g) The expressions of MAML3 in miR-486-5p transfected Jurkat and SUP-B15 cells were detected by Western blotting. (h) Jurkat and SUP-B15 cells were transfected with si-NC or si-MAML3. The relative expression level of MAML3 after transfection was detected using the qRT-PCR assay. (i) The proliferation of Jurkat and SUP-B15 was detected using CCK-8. (j) The apoptosis of Jurkat and SUP-B15 were measured using flow cytometry analysis. **P< 0.01 compared to si-NC.

MiRanda, RNA22, and TargetScan bioinformatics tools were utilized to seek the targets of miR-486-5p. In total, two potential target genes (DNAJC21 and MAML3) were found using the three tools. By using qRT-PCR assay, we observed that miR-486-5p substantially decreased MAML3 rather than DNAJC21 in both Jurkat and SUP-B15 cells (data not shown). The binding sequences between the 3-UTR of MAML3 and miR-486-5p were displayed in Figure 3(a). For the sake of confirming whether MAML3 was a downstream gene of miR-486-5p, a luciferase reporter experiment was carried out, and we found that miR-486-5p transfection weakened the luciferase activity in cells transfected with MAML3-wt luciferase reporter, whereas miR-486-5p had no action in cells transfected with MAML3-mut (Figure 3(b)). Furthermore, MAML3 was upregulated in plasma samples from patients with ALL and cells (Figures 3(c,d)). A Pearson correlation analysis suggested that the MAML3 level was negatively allied to miR-486-5p expression whereas was positively associated with PVT1 in ALL (Figures 3(e,f)). The immunoblotting assay revealed that miR-486-5p repressed the expression of MAML3 in ALL cells (Figure 3(g)). To seek the potential role of MAML3 in the growth of ALL cells, Jurkat and SUP-B15 cells were transfected with si-MAML3 (Figure 3(h)). Next, the results of CCK-8 and apoptosis assays displayed that MAML3 silencing significantly inhibited the proliferation and induced the apoptosis of ALL cells (Supplementary Figure S2 and Figures 3(i,j)).

### PVT1 is a sponge for miR-486-5p

**Figure 4.**
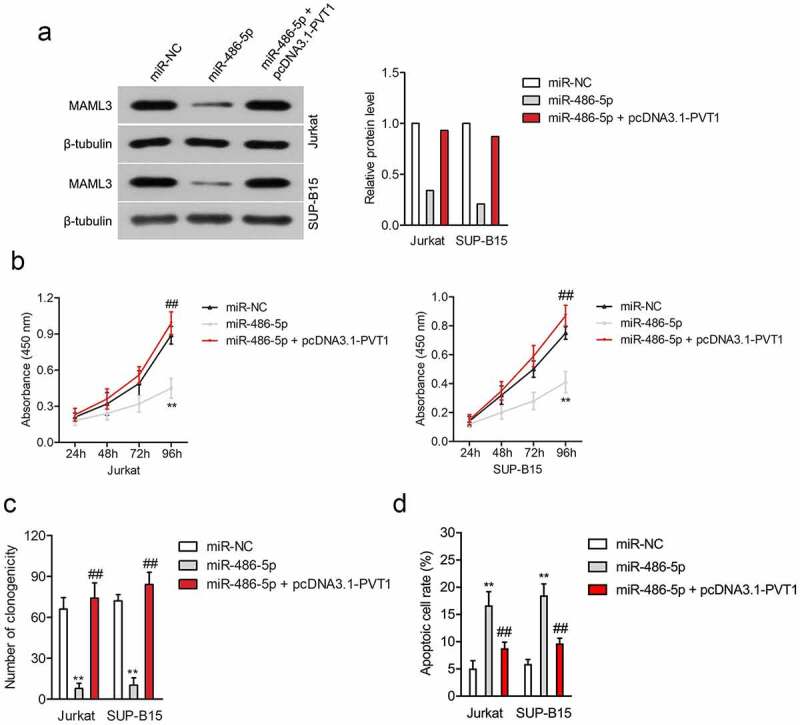
PVT1 regulated the cell viability and apoptosis of ALL cells through regulating miR-486-5p. (a) Jurkat and SUP-B15 cells were transfected with miR-NC, miR-486-5p, or miR-486-5p + pcDNA3.1-PVT1. The expression of MAML3 was determined by Western blot. (b) CCK-8 assay for transfected Jurkat and SUP-B15 cells. (c) Colony formation analysis of colony formation in Jurkat and SUP-B15 cells. (d) The apoptosis of Jurkat and SUP-B15 cells after transfection was detected using flow cytometry assay. **P< 0.01 compared to miR-NC, ^##^*P*< 0.01 compared to miR-486-5p.

To seek whether PVT1 affects ALL cells growth through acting as a sponge for miR-486-5p, pcDNA3.1-PVT1 and miR-486-5p were cotransfected into both Jurkat and SUP-B15 cells. As displayed in Figure 4(a), the inhibiting impact of miR-486-5p on the expression of MAML3 was partly neutralized by pcDNA3.1-PVT1. Next, the CCK-8 test manifested that miR-486-5p alone transfection inhibited ALL cell proliferation, whereas simultaneous cotransfection of PVT1 rescued the cell proliferation of Jurkat and SUP-B15 induced by miR-486-5p (Figure 4(b)). Consistently, transfection of miR-486-5p significantly inhibited colony formation, while upregulation of PVT1 reversed the suppressive impact of miR-486-5p (Supplementary Figure S3A and Figure 4(c)). What is more, overexpressed PVT1 offset miR-486-5p mediated induction of Jurkat and SUP-B15 cell apoptosis (Supplementary Figure S3B and Figure 4(d)).

### *PVT1 knockdown suppresses ALL cell growth* in vivo

**Figure 5.**
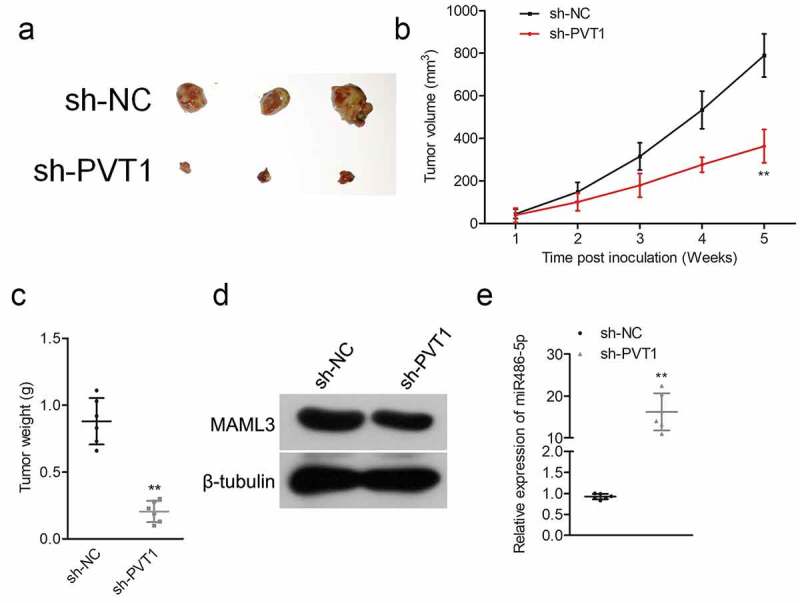
Sh-PVT1 decreased ALL cell xenograft tumor growth. (a) Representative xenograft tumor in each group. (b) Tumor volume was measured every week. (c) Tumor weight was detected in each group. (d) The protein level of MAML3 was detected in each group by Western blot. (e) The expression levels of miR-486-5p were examined in each group by qRT-PCR. **P< 0.01 compared to sh-NC.

Finally, SUP-B15 cells transfected with the sh-PVT1 or sh-NC were subcutaneously inoculated into nude mice. As shown in Figures 5(a,b), the tumor volume in mice injected with sh-PVT1 transfected SUP-B15 cells was significantly suppressed. Consistently, the weights of tumor tissues formed by sh-PVT1 transfected SUP-B15 cells were also lowered compared to that in the sh-NC group (Figure 5(c)). Moreover, we confirmed the markedly decreased MAML3 expression and elevated miR-486-5p level in tumor tissues formed by sh-PVT1 transfected SUP-B15 cells (Figures 5(d,e)). All these findings implied that PVT1 knockdown suppressed ALL cell growth *in vivo*

## Discussion

Previous investigations demonstrated that deregulated lncRNAs were associated with the progression of ALL. Early reports have demonstrated that PVT1 is significantly upregulated in peripheral blood cells from patients with APL and acute erythroleukemia (AEL) [16]. In addition, PVT1 blocked by antisense LNA GapmeRs in bone marrow cancerous blast cells could induce cell apoptosis and necrosis, which indicates that blocking PVT1 could be a potential option for the treatment of AML [17]. In ALL, PVT1 knock-down increases apoptosis rate and reduces the proliferation rate of ALL cells *in vitro* [18]. Although the function of PVT1 in ALL has been partially investigated, the underlying mechanism has not been fully illustrated. Herein, we revealed that PVT1 silencing restrained cell viability and promoted cell apoptosis of ALL cells *via* sponging miR-486-5p.

Firstly, the level of PVT1 was noticeably higher in ALL cell lines and plasma samples from ALL patients compared to control. The results from *in vitro* assays exhibited that the downregulation of PVT1 impaired ALL cells’ proliferation and colony formation. Furthermore, PTV1 silencing promoted ALL cells apoptosis. Moreover, the *in vivo* assay results manifested that PTV1 silencing inhibited the tumor formation of ALL cells in nude mice. Substantial evidence has affirmed that lncRNAs participate in ALL cells’ processes by modulating the miRNA/mRNA axis. To seek how PVT1 mediated the cell proliferation and apoptosis of ALL cells, we found that miR-486-5p had complementary sites within lncRNA PVT1 using LncBase Predicted v.2. The following luciferase reporter analysis verified the direct binding between PVT1 and miR-486-5p.

MiRNA-486-5p has been demonstrated as a fatal regulator in the development of cancers. In non-small cell lung cancer (NSCLC) and hepatocellular carcinoma, miR-486-5p serves as a suppressor [11,19]. Our results also confirmed that miR-486-5p expression level was lower in plasma specimens from ALL patients and two cell lines (Jurkat and SUP-B15) when compared within the corresponding control. Up-regulation of miR-486-5p weakened cell proliferation, clonogenicity abilities and caused ALL cells apoptosis. Notably, the expression of miR-486-5p was negatively modulated by PVT1, and its level was inversely connected to PVT1 expression in ALL.

MiRNAs are a class of critical post-transcriptional regulators that target oncogenes or cancer suppressors [20]. Then, MAML3 was verified as a downstream molecule of miR-486-5p using bioinformatics tools, which was affirmed by the luciferase reporter experiment. MAML3 is a member of the MAML family, proteins that serve as transcriptional coactivators of Notch [21]. The early report reveals that MAML3 is an oncogene, and upregulation of MAML3 induced by hypoxia increases pancreatic cancer’s growth and invasion ability [22]. Analogously, we revealed that MAML3 expression was elevated in plasma specimens from patients with ALL and two cell lines. Furthermore, the level of MAML3 was negatively regulated by miR-486-5p. Additionally, rescue assays validated that PVT1 regulated ALL cells growth and apoptotic through regulating miR-486-5p. The current study has many issues to be addressed. Although we revealed that PVT1 silencing inhibited the growth of ALL cells by sponging miR-486-5p, it might be better to use an *in vivo* model to further evaluate the interaction between PVT1 and miR-486-5p. Other biological processes, including ALL cell chemosensitivity upon PVT1 silencing, should be further explored. The results derived from 36 cases of ALL patients and 36 healthy controls are limited and need to be identified with larger sample sizes. Meanwhile, in contrast to most other studies on lncRNAs, we did not observe a significant prognostic value of PVT1 in ALL. The correlation between PVT1 expression and prognosis in ALL should be investigated in the future.

## Conclusion

Taken together, our result disclosed that PVT1 knock-down restrained cell viability and induced cell apoptosis by regulating miR-486-5p in ALL cells. MiR-486-5p negatively regulated the expression of MAML3. Moreover, PVT1 regulated cell viability and ALL cell apoptosis through sponging miR-486-5p.

## Supplementary Material

Supplemental Material
