# Supplementary material for: Long non-coding RNA (lncRNA) plasmacytoma variant translocation 1 gene (PVT1) modulates the proliferation and apoptosis of acute lymphoblastic leukemia cells by sponging miR-486-5p
Source: Bioengineered. 2022 Feb 13;13(2):4587–97. doi: 10.1080/21655979.2022.2031405 (PMC8973597; doi:10.1080/21655979.2022.2031405)
Supplement: Supplemental Material [file KBIE_A_2031405_SM4758.docx]

**
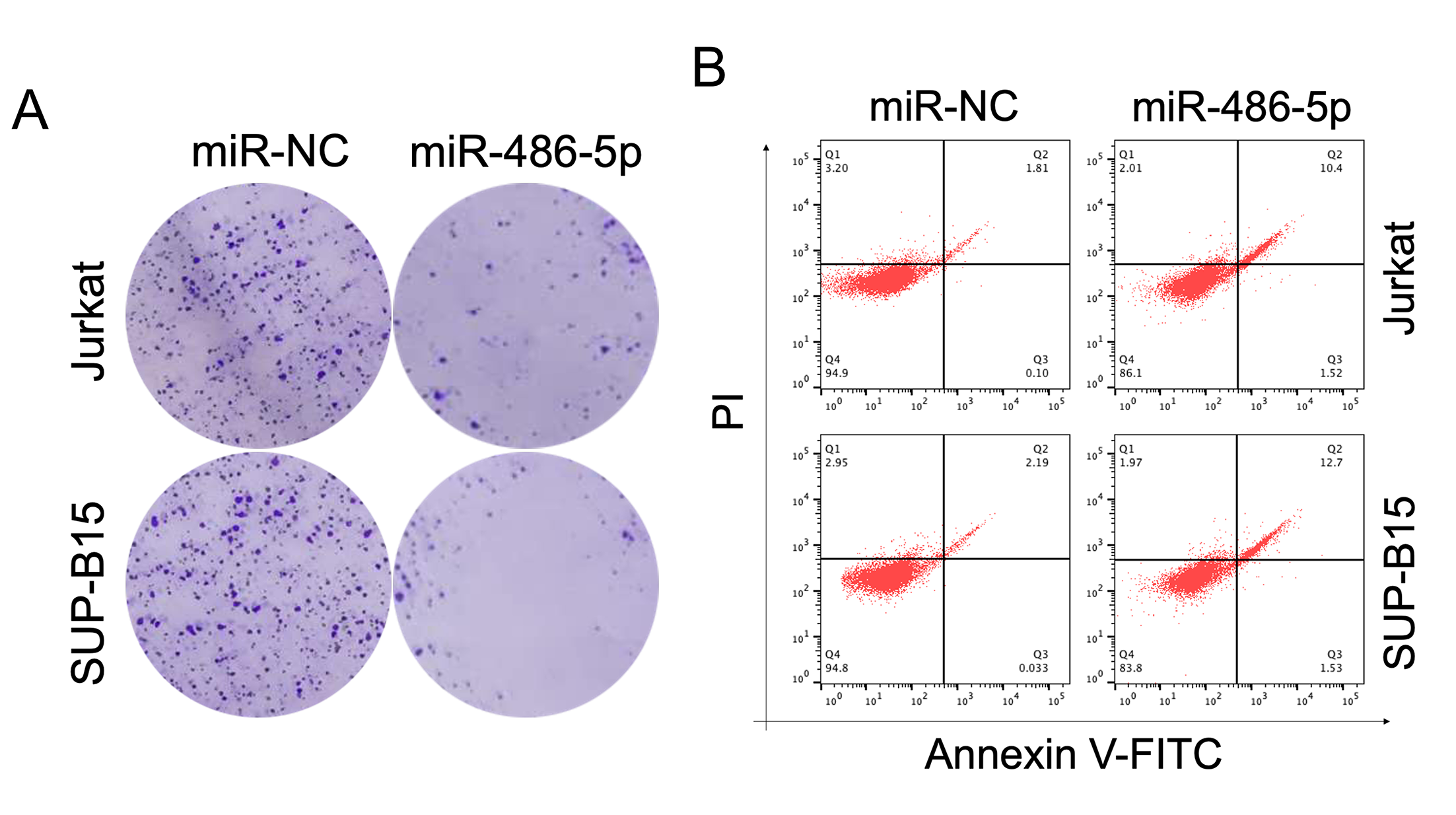
**

**Supplementary Figure 1. Upregulation of** **miR-486-5p inhibits the colony formation of ALL cells and induces cells apoptosis.** (A) MiR-486-5p inhibits ALL cells clonogenic formation. Representative pictures are shown here. (B) MiR-486-5p induces apoptosis in Jurkat and SUP-B15 cells. The representative pictures are shown for each group.

**
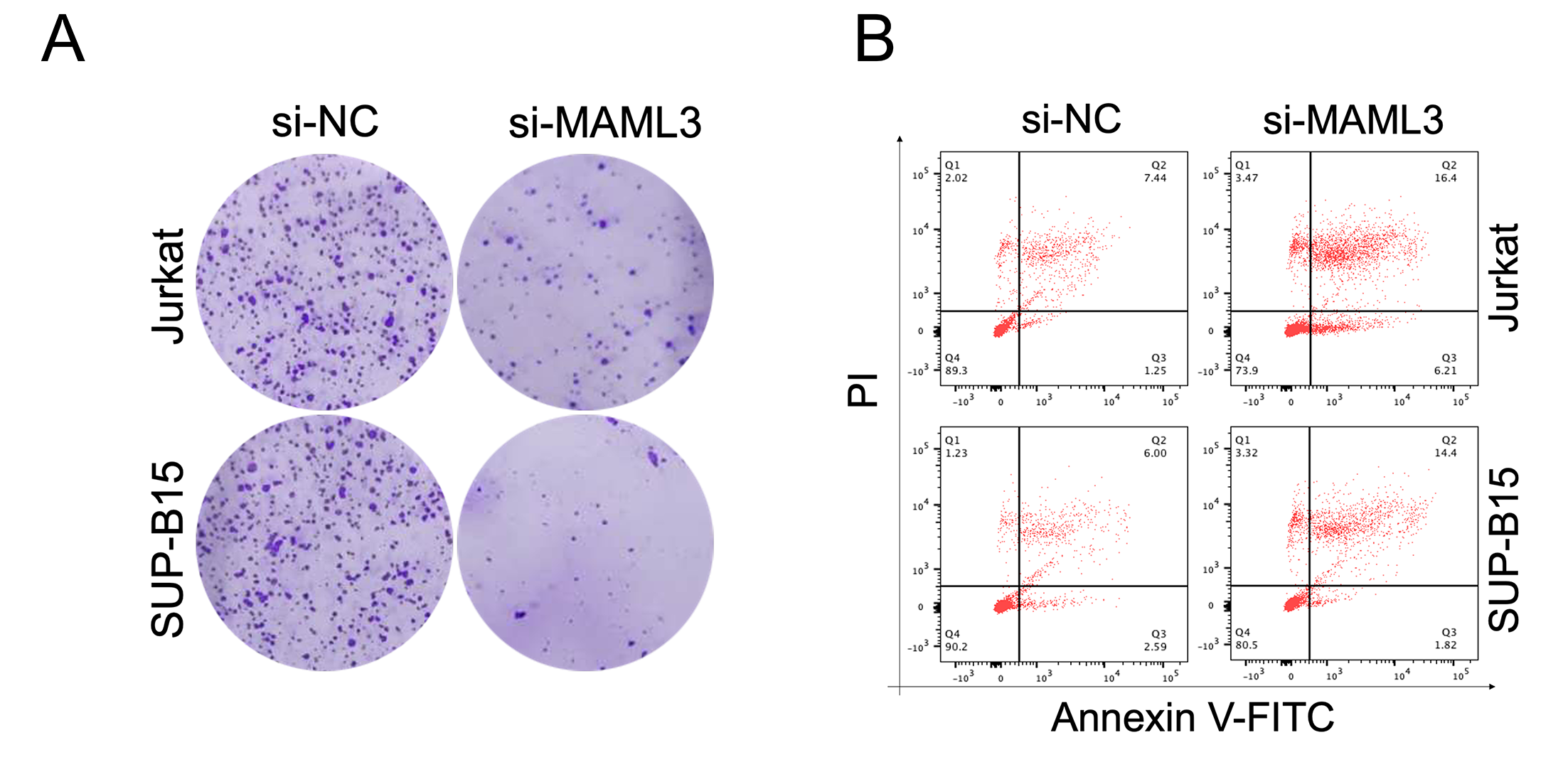
**

**Supplementary Figure 2. Deletion of MAML3 inhibits the colony formation of ALL cells and induces cells apoptosis.** (A) MAML3 knockdown inhibits ALL cells clonogenic formation. Representative pictures are shown here. (B) Silencing of MAML3 induces apoptosis in Jurkat and SUP-B15 cells. The representative pictures are shown for each group.


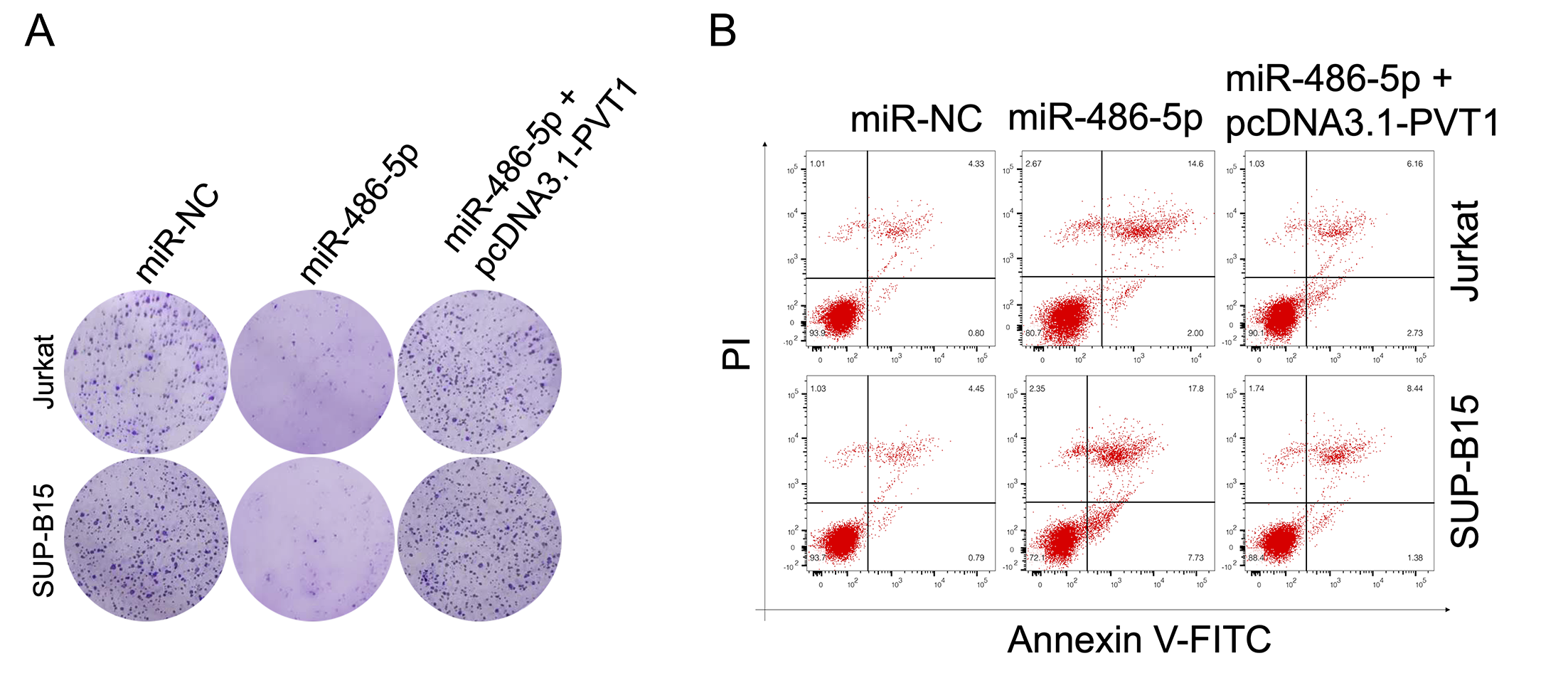


**Supplementary Figure 3. Overexpression of PVT1 reverses the inhibition of miR-486-5p.** (A) Decreased inhibition of colony formation in Jurkat and SUP-B15 cells with miR-486-5p overexpression is reversed by PVT1 overexpression. (B) Overexpression of PVT1 reverses the miR-486-5p induced effects of on Jurkat and SUP-B15 cells apoptosis.
